# Anti-Biofilm Activity: A Function of *Klebsiella pneumoniae* Capsular Polysaccharide

**DOI:** 10.1371/journal.pone.0099995

**Published:** 2014-06-16

**Authors:** Marina Dos Santos Goncalves, Cédric Delattre, Damien Balestrino, Nicolas Charbonnel, Redouan Elboutachfaiti, Anne Wadouachi, Stéphanie Badel, Thierry Bernardi, Philippe Michaud, Christiane Forestier

**Affiliations:** 1 Clermont Université, UMR CNRS 6023, Laboratoire Microorganismes: Genome Environnement (LMGE), Université d’Auvergne, Clermont-Ferrand, France; 2 Clermont Université, Université Blaise Pascal, Institut Pascal UMR CNRS 6602, Polytech Clermont-Ferrand, Aubière, France; 3 Université de Picardie Jules Verne, EA 3900-BioPI Biologie des Plantes et de l’Innovation, IUT d’Amiens (GB), Amiens cedex, France; 4 Laboratoire des Glucides FRE CNRS 3517 - Institut de Chimie de Picardie FR 3085, Université de Picardie Jules Verne, Amiens, France; 5 BioFilm Control, Biopôle Clermont-Limagne, Saint-Beauzire, France; Quuen’s University Belfast, United Kingdom

## Abstract

Competition and cooperation phenomena occur within highly interactive biofilm communities and several non-biocides molecules produced by microorganisms have been described as impairing biofilm formation. In this study, we investigated the anti-biofilm capacities of an ubiquitous and biofilm producing bacterium, *Klebsiella pneumoniae*. Cell-free supernatant from *K. pneumoniae* planktonic cultures showed anti-biofilm effects on most Gram positive bacteria tested but also encompassed some Gram negative bacilli. The anti-biofilm non-bactericidal activity was further investigated on *Staphylococcus epidermidis*, by determining the biofilm biomass, microscopic observations and agglutination measurement through a magnetic bead-mediated agglutination test. Cell-free extracts from *K. pneumoniae* biofilm (supernatant and acellular matrix) also showed an influence, although to a lesser extend. Chemical analyses indicated that the active molecule was a high molecular weight polysaccharide composed of five monosaccharides: galactose, glucose, rhamnose, glucuronic acid and glucosamine and the main following sugar linkage residues [→2)-*α*-l-Rha*p*-(1→]; [→4)-*α*-l-Rha*p*-(1→]; [*α*-d-Gal*p*-(1→]; [→2,3)-*α*-d-Gal*p*-(1→]; [→3)-*β*-d-Gal*p*-(1→] and, [→4)-*β*-d-GlcA*p*-(1→]. Characterization of this molecule indicated that this component was more likely capsular polysaccharide (CPS) and precoating of abiotic surfaces with CPS extracts from different serotypes impaired the bacteria-surface interactions. Thus the CPS of *Klebsiella* would exhibit a pleiotropic activity during biofilm formation, both stimulating the initial adhesion and maturation steps as previously described, but also repelling potential competitors.

## Introduction

Biofilms are complex assemblages of microbial cells enclosed in a self synthesized polymeric matrix [1]. They are considered as the prevalent microbial lifestyle in nature and can form biofilm on a variety of surfaces such as metals, plastics, mineral surfaces and living tissue in human host [2]–[4]. In contrast to planktonic cells, sessile cells are subjected to intense interactions due to their concentration and proximity, consisting of either cooperative or competitive phenomena [5]. These interactions can influence the emergence or the disappearance of some species within the communities and thus play important roles in the development, composition and function of the microbial consortia [6], [7]. Since biofilm formation is often considered as a major problem due to the ability of sessile bacteria to better tolerate exogenous stress than planktonic bacteria and therefore to persist, most studies have focused on antagonisms. Several bacterial non biocide biofilm-inhibiting molecules have been described so far; they impair either the initial adhesion step of the biofilm formation, its development and maturation, or the late detachment step [8]. A few anti-biofilm molecules have been isolated from monospecies biofilms, but most of them were discovered using mixed species biofilm experimental settings [9]–[11].

Indeed, mixed biofilms represent the ideal environment for discovering natural molecules that potentially influence the dynamics of bacterial populations [12]. Impairing bacterial communication systems such as the quorum sensing can affect bacterial ability to form biofilm, as in the case of *Bacillus cereus* production of an AHL lactonase inhibiting *Vibrio cholerae* biofilm settlement [13]. Cugini *et al.* (2007) also reported that *C. albicans* produces farnesol, a quorum sensing molecule, that inhibits the swarming mobility of *P. aeruginosa*, thereby enhancing its ability to form biofilm [14], [15]. Some other bacteria act differently by producing extracellular enzymes that modify the aggregates, such as the Esp protease produced by *Staphylococcus epidermidis* that degrades specific proteins in the biofilm matrix and cell wall fractions of *S. aureus* [16]. Biofilm can also be affected by the degradation of the matrix polysaccharide components. Thus Kaplan *et al.* (2003) reported that *Actinobacillus actinomycetemcomitans* produces dispersin B that degrades poly-*N*-acetylglucosamine (PNAG), a major polysaccharide component of many bacterial extracellular matrices [17].

One of the most commonly shared strategies to avoid biofilm colonization by competitors, however, consists of secretion of polysaccharides with anti-biofilm non biocide effects against other species [18]. These exopolysaccharides likely act as biosurfactants, modifying the physicochemical properties of surfaces and altering bacterial interactions within mixed biofilms [9], [19]–[22]. Thus, Valle *et al.* (2006) showed that group 2 capsules of pathogenic *Escherichia coli* reduce bacterial adhesion of commensal *E. coli* strain by inhibiting cell surface and cell-to-cell interactions in biofilm development [9]. A recent study by Rendueles *et al.* (2011) showed that commensal and pathogenic strains of *E. coli* secrete high molecular weight polysaccharides, different from capsular components, with anti-biofilm activity only against Gram-positive bacteria and able to exclude *Staphylococcus aureus* from mixed biofilms [19]. In constrast, exopolysaccharides isolated from marine *Vibrio* were shown to inhibit initial adhesion of both Gram-positive and Gram-negative by impairing both the cell to surface adherence and the bacterial intercellular adhesion [21].

In this study, we investigated the capacity of synthesis of anti-biofilm components by *Klebsiella pneumoniae*, a bacterium producing biofilms and heavily surrounded by exopolysaccharides. This work was conducted using cell-free extracts from *K. pneumoniae* and led to the isolation and characterization of a polysaccharide displaying anti-adhesion properties towards several bacterial species.

## Materials and Methods

### Bacteria and Growth Conditions

The bacterial strains used in this study are listed in table 1.

**Table 1:** Bacterial strains used in this study.

Strain	Description	Source and/or reference
***K. pneumoniae***		
MGH 78578	Capsular type K52, ATCC 700721	American Type Culture Collection
CH1031	Clinical isolate, capsular type K37	This work
NTUH-2044	Clinical isolate, capsular type K1	[44]
CF23	Clinical isolate, capsular type K1	[45]
CF7	Clinical isolate, capsular type K2	[45]
CF8	Clinical isolate, capsular type K2	[45]
LM21	Wild-type strain, capsular serotype K35	[46]
LM21 Δ*wecA*	LM21 SHV-1::*aadA7*-*gfpmut3* _*wecA*::GB Kmr Spr	[47]
LM21 Δ*wzx*	LM21 SHV-1::*aadA7*-*gfpmut3* _ORF14::GB Kmr Spr	[31]
***S. epidermidis***		
CH808	Clinical isolate	This work
RP62A	ATCC 35984, PIA +	American Type Culture Collection
CH619	CIP 68.21, PIA −	Biological Resource Center of Pasteur Institut
CH886	Clinical strain, PIA +	This work
***S. aureus***		
CH726	CIP 107422, PIA −	Biological Resource Center of Pasteur Institut
CH937	Clinical isolate	This work
CH939	Clinical isolate	This work
15981	Clinical isolate, PIA +	[48]
***P. aeruginosa***		
CH1204	Clinical isolate	This work
CH1205	Clinical isolate	This work
***E. coli***		
TG1	K-12 laboratory strain	
***P. mirabilis***		
CH1063	Clinical isolate	This work
***L. monocytogenes***		
EGD	Wild-type	[49]
***E. aerogenes***		
CH716	Clinical isolate	This work
***B. cereus***		
ATCC 12826	Type strain A, variant IV	American Type Culture Collection

All bacterial strains were stored at −80°C in Trypticase Soy (TS) broth containing 15% glycerol (v/v). They were subcultured from freezer stocks onto TS agar plates (TSA, Fisher Scientific) or in M63B1 broth supplemented with glucose (0.4% (w/v)). All subsequent liquid precultures (overnight cultures) were performed at 37°C in TS broth (TSB, Oxoid, Basingstoke, England) at 200 rpm in an orbital shaking and were derived from colonies isolated on TSA plates. For planktonic growth cultures, *S. epidermidis* RP62A and *K. pneumoniae* MGH 78578 were cultured in TSB at 37°C in aerobic conditions. Results of *S. epidermidis* numeration were expressed as the number of CFU.mL^−1^ of suspension. For quantification of capsular polysaccharides, *K. pneumoniae* strains were cultivated overnight in D.W. medium supplemented with 0.1% Casamino Acids, 200 µg of MgCl_2_ per ml, 20 µg of CaCl_2_ per ml, 1 µg of ZnCl_2_ per ml, and 4 µg of FeCl_3_ per ml [23].

### *K. pneumoniae* Cell-free Extracts

Cell free supernatants from *K. pneumoniae* planktonic bacteria were obtained from 24 h-old cultures centrifuged for 10 min at 7,000×*g* at 4°C and the supernatants were filtered through a 0.2 µm filter (CA-membrane, Sartorius Stedim, North America).

Biofilm cell free extracts, i.e. biofilm supernatant and acellular matrix material, were prepared from biofilms grown in tissue culture dish (Falcon, Becton Dickinson, Franklin Lakes, USA) using an inoculum of 1×10^6^ CFU.mL^−1^ and incubated for 24 h at 37°C. The biofilm supernatants were recovered as described above for the planktonic supernatant. For acellular matrix material, the biofilms were then lifted by scraping, sonicated in a waterbath sonicator for 5 min in order to dissociate bacterial aggregates and thus free the extracellular material, vortexed and again sonicated for 5 min. To harvest the resulting 2 mL biofilm acellular material, the suspensions were centrifuged (7,000×*g* for 10 min) and filtered throughout a 0.2 µm filter.

### Effect of *K. pneumoniae* Cell-free Extracts on Biofilm Formation

The effects of the three types of *K. pneumoniae* MGH 78578 extracts (planktonic supernatant, biofilm supernatant and acellular matrix biofilm) were assessed on biofilm formation using the three following procedures. To determine the spectrum of activity of the *K. pneumoniae* anti-biofilm component, biofilm formation assay was carried out in 96-well polystyrene microtiter plates (Falcon) and measured using the crystal violet staining procedure. Briefly, overnight cultures of each strain to be tested were diluted to about 1×10^6^ CFU.mL^−1^ with fresh TSB. Each well of the microtiter plates was filled with inoculum of the bacterial suspensions containing ¼ (v/v) of *K. pneumoniae* MGH 78578 supernatant extract (concentration 2.654 mg equivalent galactose per milliliters). Control assays were performed by forming biofilm without any supernatant extract. The microtiter plates were incubated at 37°C for 8 h without shaking, and non-adherent bacteria removed by three washings with saline solution (9‰ NaCl (w/v)). Biofilm was stained by 0.5% (w/v) crystal violet solution for 10 min. Then, the plates were rinsed under running tap water, air-dried, and the crystal violet was resuspended in ethanol, and the OD_570_ was determined. The composition of the biofilm matrix of the *Staphylococcus* strains tested (polysaccharide or protein) was assessed essentially as described in Wang *et al.* (2004), by treatment with proteinase K or sodium *meta*-periodate [24].

Biofilms were also measured using a magnetic beads-mediated agglutination assay measuring the immobilization of magnetic beads embedded in bacterial aggregates following biofilm formation (Ring test®, Biofilm Control, Saint-Beauzire, France). Consequently, the more beads are entrapped by cells, the less they are detectable after magnetization [25]. Three µm diameter magnetic particles (toner) were added to bacterial suspensions obtained after serial dilutions in TSB of an overnight culture or to TSB alone (control solution) at a final concentration of 10 µL.mL^−1^. Each well was then inoculated with 200 µL of the mixture containing either *S. epidermidis* RP62A at concentration of 1×10^6^ CFU.mL^−1^ with or without (control) ¼ (v/v) of either *K. pneumoniae* planktonic supernatant, biofilm supernatant or acellular matrix material. Three experiments with three repeats each were performed. After incubation at 37°C, 100 µL of contrast liquid (inert oil) was added to each well and the strip wells were immediately scanned and placed for 1 min on the block test for magnetization and re-scanned. This test is based on the concept of immobilization of beads by sessile bacteria that form aggregates with enough strength to overcome the magnetic attraction forces applied on them. Thus, in the absence of sessile cells all the beads gathered in the center of the wells and formed an easily detectable black spot. Images of each well before (I_0_) and after (I_1_) magnetization were compared with the Biofilm Control® Software and the discrepancies between the two images gave rise to values named BioFilm Index (BFI) ranging from 0 to 30. High BFI values correspond to high mobility of beads under magnet action and therefore little or no immobilization of the beads by bacteria [25]. With TSB as incubation medium, a biofilm was considered installed when the value of BFI was less than or equal to 4.5, which corresponds to 10% untrapped beads.

For CFU determination, biofilms were formed in duplicate on Thermanox® slides (Nalgène) incubated at 37°C in 24-well plates (Falcon, Becton Dickinson, Franklin Lakes, USA) in TSB for 8 hours. Coverslips were then gently removed and washed twice with sterile physiological water (9‰ NaCl (w/v)). Adhering bacteria were resuspended in 2 mL of physiological water and then sonicated three times for 5 minutes (S-LINE FisherBrand, 37 kHz) and vortexed between each sonication. The resulting biofilm suspensions were then serial diluted and plated onto selective agar media to determine the numbers of CFU.

### Electron Microscopic Observation

For scanning electronic microscopy observation, biofilms were formed on Thermanox® slides as described above and fixed overnight at 4°C with a solution of 0.2 M cacodylate buffered at pH 7.4 supplemented with glutaraldehyde at 1.6% (w/v) and ruthenium red at 0.05% (w/v). They were then rinsed in the same buffer. After post-fixation for 1 h with osmic acid in cacodylate buffer, biofilms were dehydrated using a graded ethanol series (10 min each in 25%, 50%, 75%, 100% absolute alcohol), and 100–150 mL of hexamethyldisilazine were added to each insert and allowed to evaporate (∼2 h) in a fume hood. Supports were attached to 12 mm diameter aluminium SEM stubs using adhesive carbon tabs and they were gold coated using a sputter coater. The specimens were examined by a JSM-6060LV scanning electron microscopy (JEOL, Croissy-sur-Seine, France).

### Bacterial Hydrophobicity Measurement

The Microbial Adhesion To Solvents (MATS) test was performed to evaluate the Lewis acid-base properties and the hydrophilic/hydrophobic nature of bacterial surfaces. This test was adapted from the method of Rosenberg *et al.* (1980) which is based on the adhesion of bacterial cells to four different solvents: chloroforme, hexadecane, decane and ethyl acetate [26]. 10 mL of bacteria grown overnight in LB were harvested by centrifugation (5,000×*g*, 8 min), washed twice with sterile PBS and resuspended in 10 mL of PBS. The OD_600nm_ of the suspension was measured and adjusted at 0.4 (OD_600nm_ initial). One mL of solvent was mixed to 3 mL of the cell suspension and vortexed for 1 min. The two phases were allowed to separate for 20 min. One mL of the aqueous phase was carefully removed and the OD_600nm_ was measured (OD_600_ final). The microbial adhesion to each solvent was calculated using the formula: % affinity = 100×(1−(OD_600_ final/OD_600_ initial). Each experiment was performed in triplicate.

### Physical and Chemical Analyses of *K. pneumoniae* Planktonic Extract

For enzymatic treatments, planktonic extract was incubated for 1 h at 37°C with 100 mg.L^−1^ DNase I, RNase A, porcine pancreatic lipase or proteinase K (Sigma-aldrich). Controls consisted of mock-treated extracts, or enzyme alone without any extract. For sodium metaperiodate treatment, 0.1 vol of 100 mM sodium metaperiodate was added to the extract, and incubated at 37°C for 1 h. Controls consisted of mock-treated extract and sodium metaperiodate alone. Following all treatments, extracts and controls were incubated at 100°C for 10 min prior to testing in the *S. epidermidis* biofilm assay as described above. Temperature influence itself was assessed by heating the supernatant extract for 10 min at 100°C of without any other treatment.

### Extraction, Purification and Chemical Characterization of the anti-biofilm Compound

#### (1) Production and extraction of anti-biofilm compound

*K. pneumoniae* MGH 78578 was incubated for 6 h at 37°C in TSB. The culture was then centrifuged at 7,000×*g* for 10 min and the pellet was washed twice with M63B1 minimum medium. The bacteria were resuspended in M63B1 supplemented with glucose (0.4% w/v). After two washes in this medium, cells were incubated for 24 h at 37°C with orbital shaking. Cultures were centrifuged for 10 min at 7,000×*g* at 4°C and the supernatants were filtered through a 0.2 µm filter (Stericup, millipore).

The supernatant was concentrated by evaporation with rotary evaporator (Heidolph Laborota 4000 Efficient) coupled to a vacuum pump (ILMVAC LVS 210 T) and extensively dialysed (CelluSep, H1 membrane, 3 kDa cut-off) with gentle agitation at 4°C during 8 days against ultrapure water (3 baths/day).

#### (2) Monosaccharide composition determination

Monosaccharide composition of polysaccharides was evaluated by High Pressure Anion Exchange Chromatography (HPAEC) on an ICS 3000 (Dionex, USA) equipped with pulsed amperometric detection and AS 50 autosampler. It was assembled with a guard CarboPac PA1-column (4×50 mm) and analytical CarboPac PA1-column (4×250 mm). Before analysis, the dried extract from *K. pneumoniae* MGH 78578 planktonic extract was hydrolyzed in 4 M TFA for 8 h at 100°C and neutralized in 4 M NH_4_OH. Samples (10 mg.mL^−1^) were filtered using 0.2 µm membrane filter and injection volume was fixed at 25 µL. Before each injection, columns were equilibrated by running during 15 min with 18 mM NaOH. Samples were eluted isocratically with 18 mM NaOH for 30 min, followed by a linear gradient between 0 to 1 M sodium acetate in 200 mM NaOH for 20 min to elute acidic monosaccharides. Run was followed by 15 min washing with 200 mM NaOH. The eluent flow rate was kept constant at 1 mL.min^−1^. Columns were thermostated at 25°C. Data were collected and analyzed with Dionex Chromeleon 6.80 software (Sunnyvale, USA).

#### (3) SEC-MALLS analysis

Average molecular weights and molecular weight distributions were determined by high pressure size exclusion chromatography (HPSEC) with on line multi-angle laser light scattering (MALLS) filled with a K5 cell (50 µL) and two detectors: a He–Ne laser (*λ* = 690 nm) and a differential refractive index (DRI). Columns [OHPAK SB-G guard column, OHPAK SB806, 804 and 803 HQ columns (Shodex)] were eluted with NaNO_3_ 0.1 M at 0.7 mL.min^−1^. Solvent was filtered through 0.1 µm filter unit (Millipore), degassed and filtered through a 0.45 µm filter upstream column. The sample was injected at 5 g.L^−1^ through a 100 µL full loop. The collected data were analyzed using the Astra 4.50 software package and a dn/dc of 0.15.

#### (4) Size Exclusion Chromatography (SEC)

The planktonic extract from *K. pneumoniae* MGH 78578 at 10 g.L^−1^ in 50 mM phosphate buffer (pH 7) supplemented with NaCl (150 mM) was filtered (at 0.45 µm) and analyzed by SEC at room temperature. The column used was a Superdex 200 column (1.5 cm×50 cm), (GE Healthcare, Sweden) coupled to an ÄKTA Purifier system (Amersham Pharmacia Biotech, Sweden) and eluted with a 50 mM phosphate buffer (pH 7) supplemented with NaCl (150 mM) at a flow rate of 0.5 mL.min^−1^. Fractions of 0.5 mL were collected and the sugar content was determined by phenol–sulphuric acid method. Each fraction collected was assayed by the magnetic beads-mediated agglutination assay to determine the anti-biofilm activities with dialyzed planktonic extract from *K. pneumoniae*.

#### (5) NMR analysis

The dried polysaccharide was dissolved in D_2_O (99.9% D) and freeze-dried to replace exchangeable protons with deuterium. For NMR analysis, the exchanged polysaccharide was dissolved in D_2_O at (40 g.L^−1^). The NMR spectra of the solutions were recorded at 60°C using a Brucker Advance 600 spectrometer of 300 MHz equipped with ^13^C/^1^H dual probe. The NMR experiments were recorded with a spectral width of 3000 Hz, an acquisition time of 1.36 s, a pulse width of 7 µs, a relaxation time of 1 s and a number of 256 scans. The HOD signal was presaturated by a presaturation sequence.

#### (6) Total sugar quantification

The quantity of total polysaccharides was assessed by the phenol sulphuric acid method [27] using galactose as standard.

### Surface Coating Assay with Capsular Polysaccharide Extract from *K. pneumoniae* Strains

Capsular polysaccharides were extracted from bacteria grown overnight in DW, according to the method previously described by Domenico *et al.* in 1989 [23]. Briefly, 500 µL of bacterial cultures were mixed with 100 µL of 1% Zwittergent 3–14 detergent (w/v) (fluka, chemika) in 100 mM citric acid. This mixture was incubated at 56°C for 20 min. After centrifugation for 5 min at 20,000×*g*, 300 µL of the supernatant was transferred to a new tube and 1.2 mL of absolute ethanol was added. The mixture was placed at 4°C for 20 min. After centrifugation, the supernatant was decanted and the pellet was washed once with 70% ethanol, and then dissolved in 250 µL of distilled water.

A volume of 25 µL of capsular polysaccharide extract of *K. pneumoniae* strains (with equivalent quantities of CPS) or 25 µL of PBS as a control, was transferred to the center of a well of a 24-well tissue-culture-treated polystyrene microtiter plate (Falcon no. 353047). The plate was incubated for 48 h at 4°C. The excess of extract was removed by pipetting and the plate was incubated at room temperature to allow complete evaporation of the liquid. The wells were then filled with 1 mL of TS broth containing 1×10^6^ CFU.mL^−1^ of *S. epidermidis* RP62A. After 8 h, wells were rinsed with water and stained with 1 mL of 0.5% (w/v) crystal violet solution. Then, the wells were rinsed under running tap water, air-dried and were photographed.

### Statistical Analysis

For analysis of the significance of differences in bacterial biomass between biofilm and planktonic culture, two-tailed Student’s *t*-tests were used to compare two groups of data. All experiments were done at least three times. A *P*-value of ≤0.05 was considered to be statistically significant.

## Results

### Influence of *K. pneumoniae* Cell-free Extracts on Biofilm Formation

**Figure 1:**
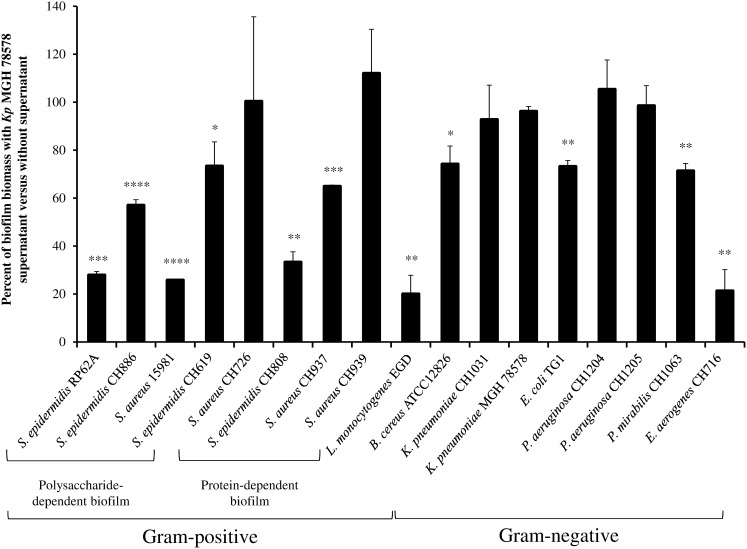
Anti-biofilm activity of *K. pneumoniae* polysaccharide against several bacterial species. Results are expressed as the percentages of biomass in presence of polysaccharides extract *versus* without any extract (control), as determined by crystal violet staining. Experiments were performed in triplicate; error bars represent standard deviations. Statistical *t* test was used to evaluate the significance of growth inhibition. *, p-value<0.05; **, p-value<0.01; ***, p-value<0.001.

The anti-biofilm activity of *K. pneumoniae* supernatant was assessed on several bacterial species and quantification of the biofilm biomass performed by crystal violet staining indicated no significant effect on *K. pneumoniae, P. aeruginosa* biofilms and some *S. aureus* isolates. In contrary, the biomass of *E. coli*, *S. epidermidis*, *P. mirabilis*, *E. aerogenes*, *B. cereus* and *L. monocytogenes* biofilm were significantly reduced in the presence of *K. pneumoniae* supernatant extract (Fig. 1). All the *Staphylococcus* strains forming polysaccharide-dependent biofilm were inhibited by *K. pneumoniae* supernatant extract, whereas only two strains with protein-dependent biofilm characteristic were impaired (Fig. 1). Microbial adhesion to solvents method (MATS) was performed on two of the *S. aureus* strains forming protein-dependent biofilm CH937 (affected by supernatant) and CH939 (unaffected). Results indicated that the CH937 strain had an acid and basic character while the non-susceptible isolate, CH939, harboured a more basic surface (data not shown), a difference that might account in the interactions with the supernatant active molecules.

**Figure 2:**
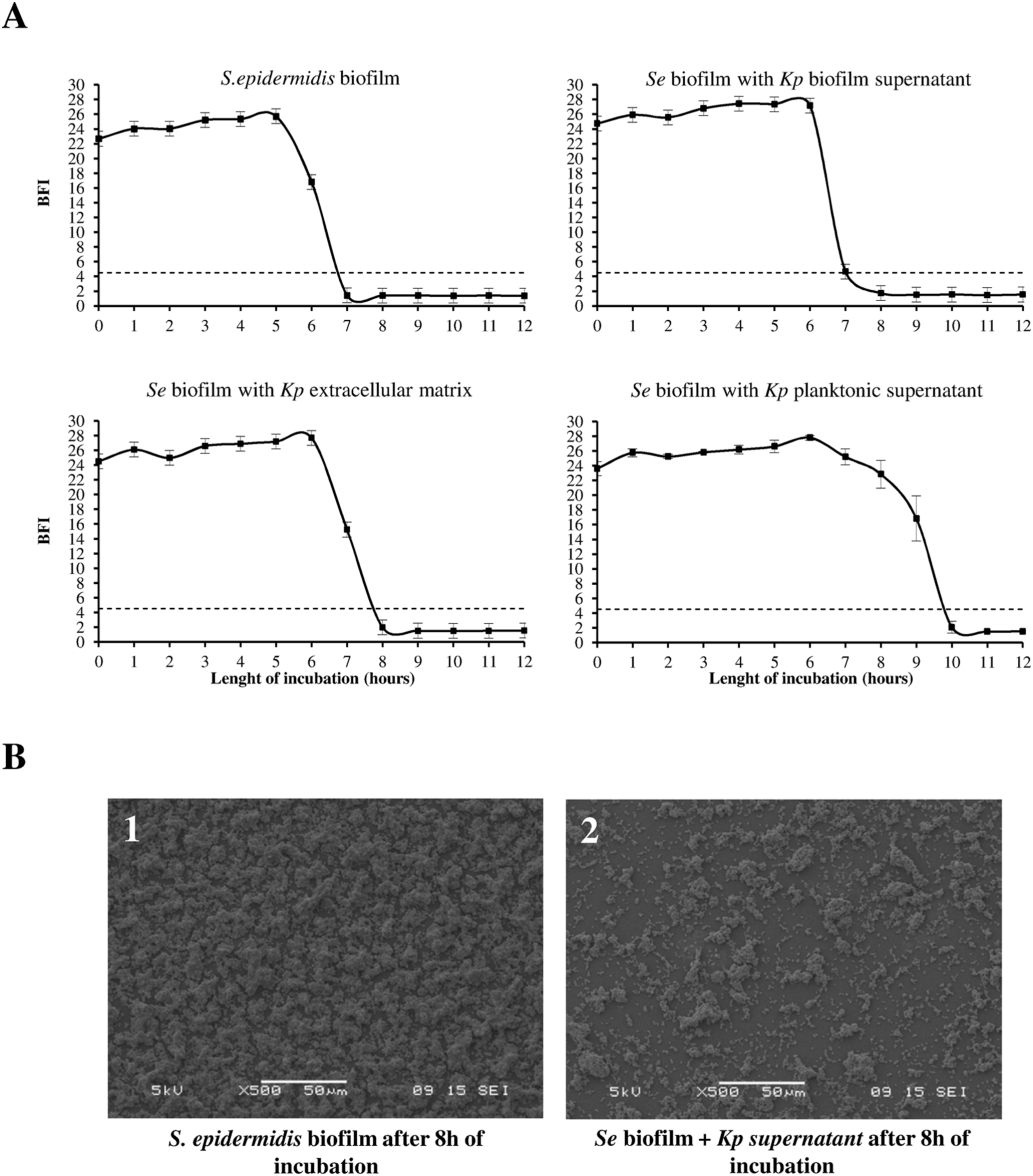
Effect of planktonic, biofilm and extracellular matrix extracts of *K. pneumoniae* on S. *epidermidis* biofilm formation. *Se* (inoculum, 10^6^ CFU/mL) biofilm formation on polystyrene microtiter plates measured with BioFilm Magnetic bead aggregation assay®. (A) Quantitative data expressed as BFI (means ± SD of three determinations). The dotted line represents the threshold of magnetic bead-mediated agglutination detection. (B) SEM observation of monospecies biofilms of *S. epidermidis* (inoculum, 10^6^ CFU.mL^−1^) formed in presence or absence of *K. pneumoniae* supernatant on Thermanox® slides after 8h of incubation. *S. epidermidis* biofilm: (1) without *K. pneumoniae* supernatant, (2) with *K. pneumoniae* supernatant.

To determine if the effect of *K. pneumoniae* was due to some released molecules specifically produced in planktonic growth conditions, we investigated the effect of two other types of *K. pneumoniae* cell-free extracts, biofilm supernatant and acellular matrix biofilm, on the adhesive capacity of *S. epidermidis* RP62A, one of the highest attenuated strain. Monitoring of biofilm formation was performed over time using a magnetic beads-mediated agglutination assay. The addition of all cell free extracts from *K. pneumoniae* cultures modified biofilm installation by *S. epidermidis*, but the effect was more pronounced with planktonic supernatants (Fig. 2A). In addition, experiments carried out with serial dilutions of planktonic supernatant and *S. epidermidis* RP62A showed that the inhibition of agglutination was dose-dependent (Fig. S1).

SEM observations of *S. epidermidis* biofilm formed in the presence of *K. pneumoniae* planktonic supernatant showed small clusters dispersed on the surface, in contrast to the dense aggregates observed in the control (without the extract) (Fig. 2B). Similar effects were observed using supernatant extracts from other *K. pneumoniae* isolates (data not shown), indicating that this anti-biofilm effect was not strain-dependent.

**Figure 3:**
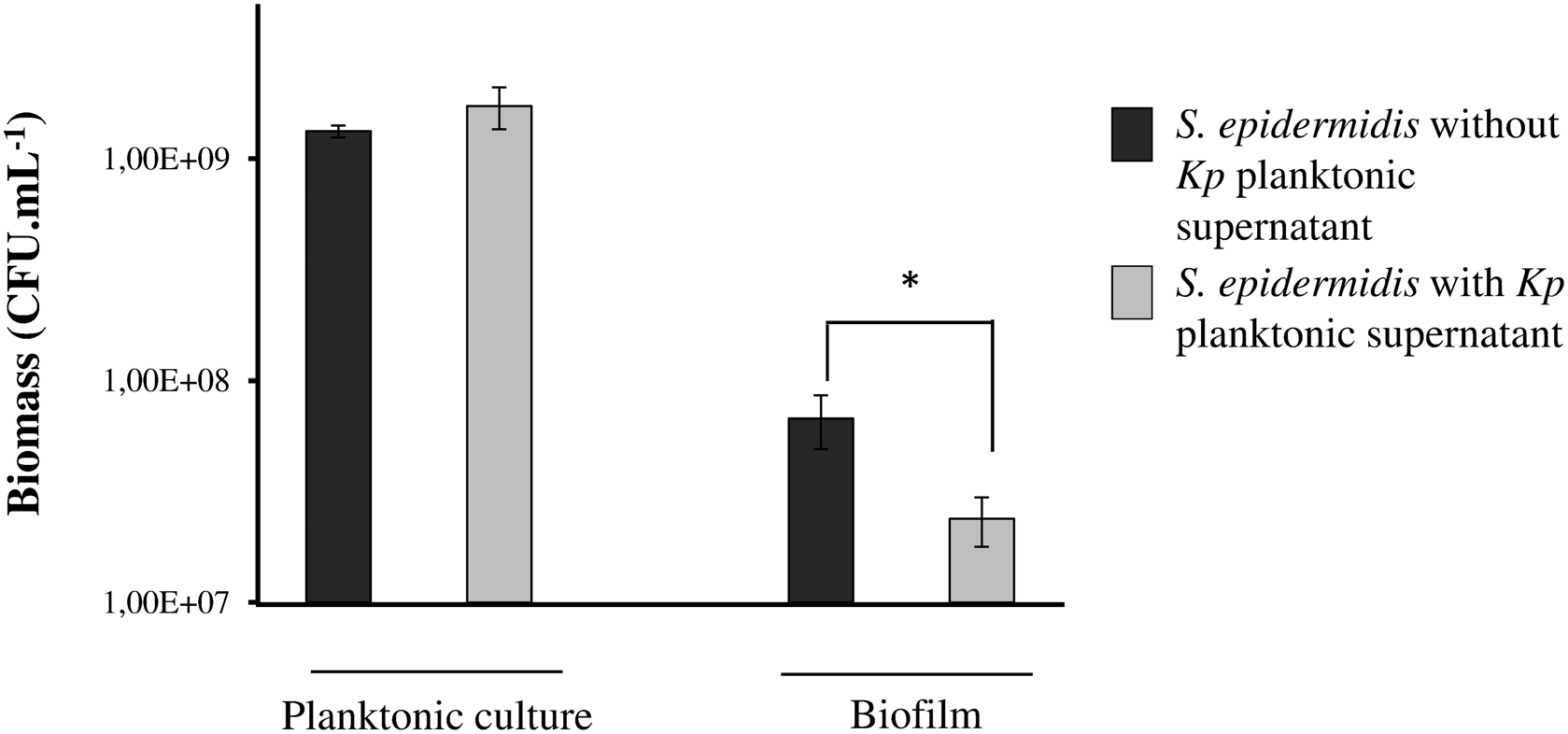
Effect of *K. pneumoniae* planktonic supernatant on *S. epidermidis* biomass. Quantification of viable bacteria was performed in planktonic and biofilm cultures, with and without addition of *K. pneumoniae* planktonic supernatant extract. Experiments were performed in triplicate; error bars represent standard deviations. Statistical *t* test was used to evaluate the significance of growth inhibition. *, p-value<0.05.

Determination of the biomass indicated that the number of *S. epidermidis* CFU in the biofilms recovered after 8 hours of incubation was significantly lower in the presence of planktonic supernatants of *K. pneumoniae* (2.39×10^7^ CFU.mL^−1^) compared to the control (*S. epidermidis* alone (6.77×10^7^ CFU.mL^−1^) (p<0.05), whereas no effect was observed on the growth of *S. epidermidis* in planktonic cultures (Fig. 3). Consequently, the lower number of CFUs observed in biofilms treated with *K. pneumoniae* supernatant was probably due to an anti-adhesion effect rather than a biocide effect.

### Physico-chemical Characterization

**Figure 4:**
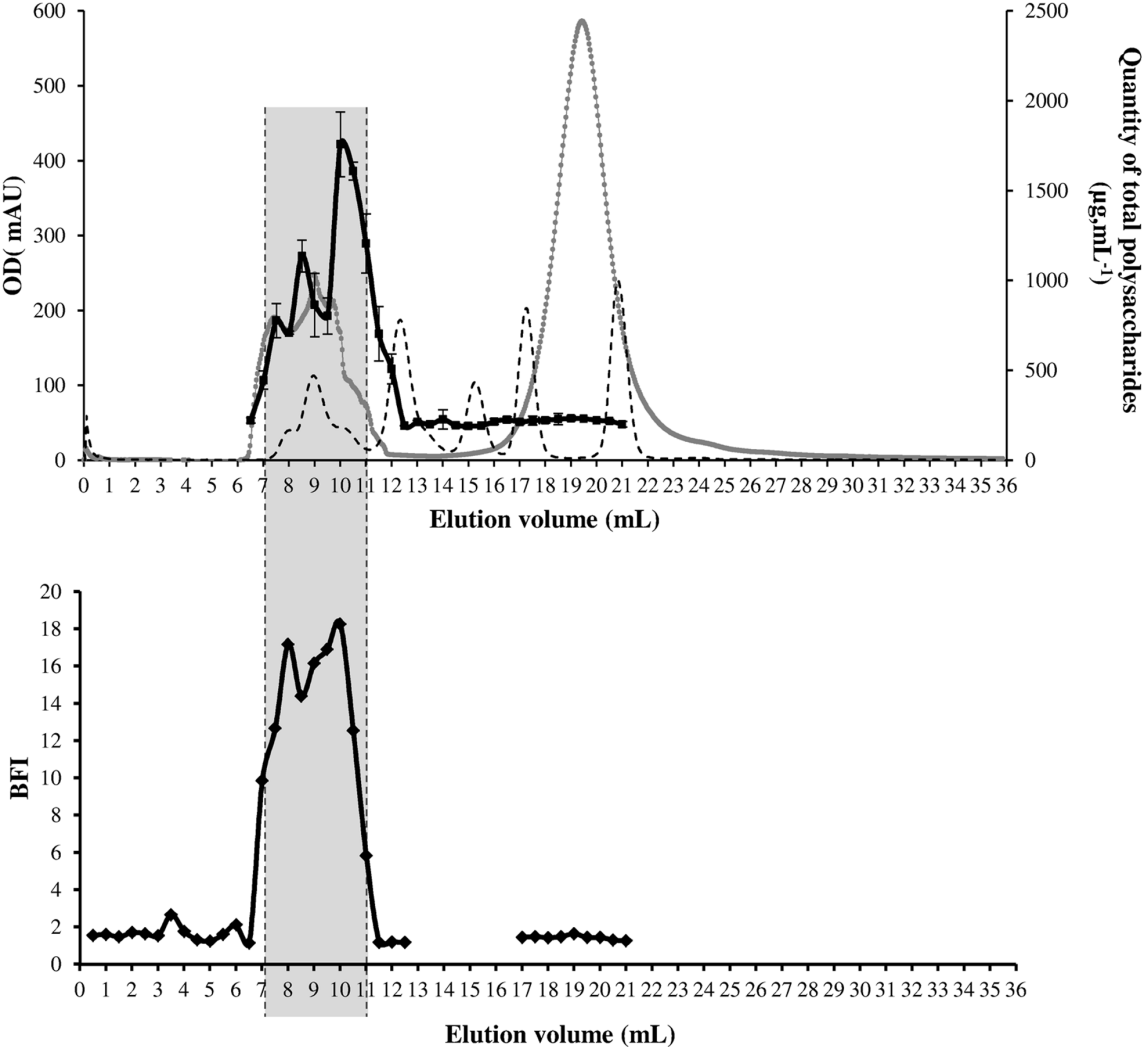
Identification of the active fractions in planktonic supernatant extract. Size exclusion chromatograph of planktonic extract from *K. pneumoniae* together with quantification of total polysaccharides assessed by the phenol sulphuric acid method were performed. Elution profile of standard markers (tyroglobulin, γ-globulin, ovalbumin, myoglobulin, vitamin B12, respectively) is represented by the dotted curve. Anti-biofilm activity of each sample was monitored by magnetic bead-mediated agglutination assays and expressed as Biofilm Formation Indices (BFI). Only samples collected between 7 and 11 mL displayed anti-biofilm activity (gray area).

**Figure 5:**
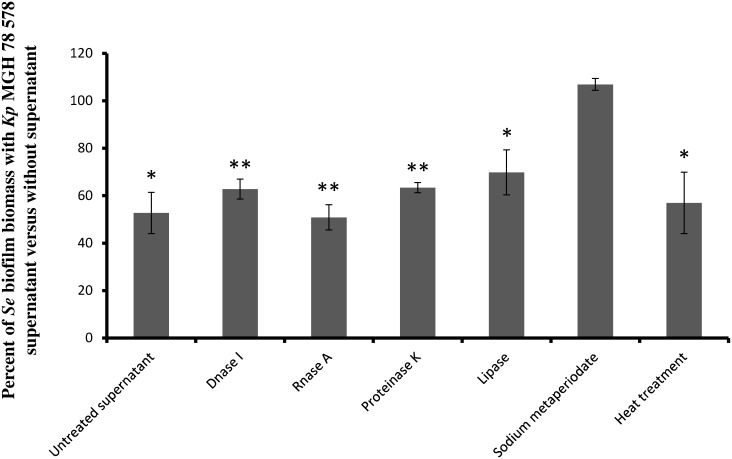
Physico-chemical analysis of the component responsible for the anti-biofilm activity in *K. pneumoniae* supernatant. The *K. pneumoniae* supernatant was treated with Proteinase K, lipase, DNase I, RNase, sodium metaperiodate or by heat, and the anti-biofilm activity against *S. epidermidis* was measured by crystal violet staining. Results are expressed as percentages of biomass obtained with treatment *versus* without any treatment. Experiments were performed in triplicate; error bars represent standard deviations. Statistical *t* test was used to evaluate the significance of growth inhibition. *, p-value<0.05; **, p-value<0.01.

Preliminary analysis of *K. pneumoniae* supernatant by size exclusion chromatography (SEC) using a Superdex 200 column indicated that the active fraction (samples between 7 and 11 mL of elution volume) contained compounds with molecular weight higher than 100 kDa (Fig. 4). Treatments of the *K. pneumoniae* supernatant with proteinase K, lipase, DNase or RNase or heat did not impair the capacity of *S. epidermidis* to form biofilm (Fig. 5). In contrast, treatment with carbohydrate-active agent sodium metaperiodate significantly reduced its anti-biofilm activity (Fig. 5), suggesting that the anti-biofilm activity was due to heat stable macromolecules mainly composed of sugars (polysaccharide).

### *K. pneumoniae* Capsular Polysaccharide Cell-to-surface Interactions

**Figure 6:**
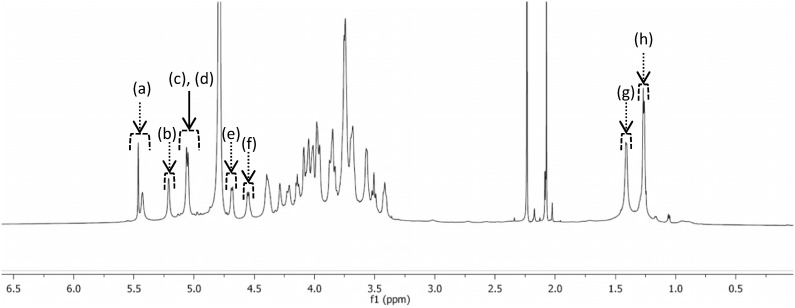
^1^H NMR analysis of dried extract from *K. pneumoniae* supernatant at 40 g.L^−1^ in D2O (99.99% D). Analysis was recorder at 60°C using a Brucker Advance spectrometer (600 MHz). (a) = H-1 of [→2,3)-*α*-d-Gal*p*-(1→] residue; (b) = H-1 of [*α*-d-Gal*p*-(1→] residue; (c) = H-1 of [→4)-*α*-l-Rha*p*-(1→] residue; (d) = H-1 of [→2)-*α*-l-Rha*p*-(1→] residue; (e) = H-1 of [→4)-*β*-d-GlcA*p*-(1→] residue; (f) = H-1 of [→3)-*β*-d-Gal*p*-(1→] residue; (g) = H-6 of [→4)-*α*-l-Rha*p*-(1→] residue; (h) = H-6 of [→2)-*α*-l-Rha*p*-(1→] residue.

The molecular weight of this polysaccharide was evaluated by SEC-MALLS analysis at 1.5×10^6^ Daltons and the low polydispersity index (1.3) confirmed the presence of polysaccharide macromolecule family. Further HPAEC analysis after acid hydrolysis of extracted and partially purified polysaccharide of *K. pneumoniae* indicated that it was composed of five monosaccharides: galactose (47.8%), glucose (27.6%), rhamnose (15.4%), glucuronic acid (6.1%) and glucosamine (3.1%) and an ^1^H NMR analysis showed 6 specifics resonance peaks (Fig. 6) corresponding to the mains following sugar linkage residues [→2)-*α*-l-Rha*p*-(1→]; [→4)-*α*-l-Rha*p*-(1→]; [*α*-d-Gal*p*-(1→]; [→2,3)-*α*-d-Gal*p*-(1→]; [→3)-*β*- d-Gal*p*-(1→] and, [→4)-*β*- d-GlcA*p*-(1→] (Table 2).

**Table 2:** ^1^H NMR analysis of mains sugar linkage residues of polysaccharide fraction extracted from *Klebsiella pneumoniae*.

Sugar linkage residue	Chemical shift (δppm)					
	H-1	H-2	H-3	H-4	H-5	H-6
→2)-*α*-l-Rha*p*-(1→	5.05	4.05	3.83	3.51	4.01	1.27
→4)-*α*-l-Rha*p*-(1→	5.06	4.09	4.02	3.69	3.98	1.41
* α*-d-Gal*p*-(1→	5.21	3.85	3.96	4.03	4.31	3.74
→2,3)-*α*- d-Gal*p*-(1→	5.45	4.22	4.14	4.29	4.33	3.73
→3)-*β*- d-Gal*p*-(1→	4.58	3.75	3.74	4.05	3.75	3.88
→4)-*β*- d-GlcA*p*-(1→	4.71	3.42	3.57	3.68	3.76	-

**Figure 7:**
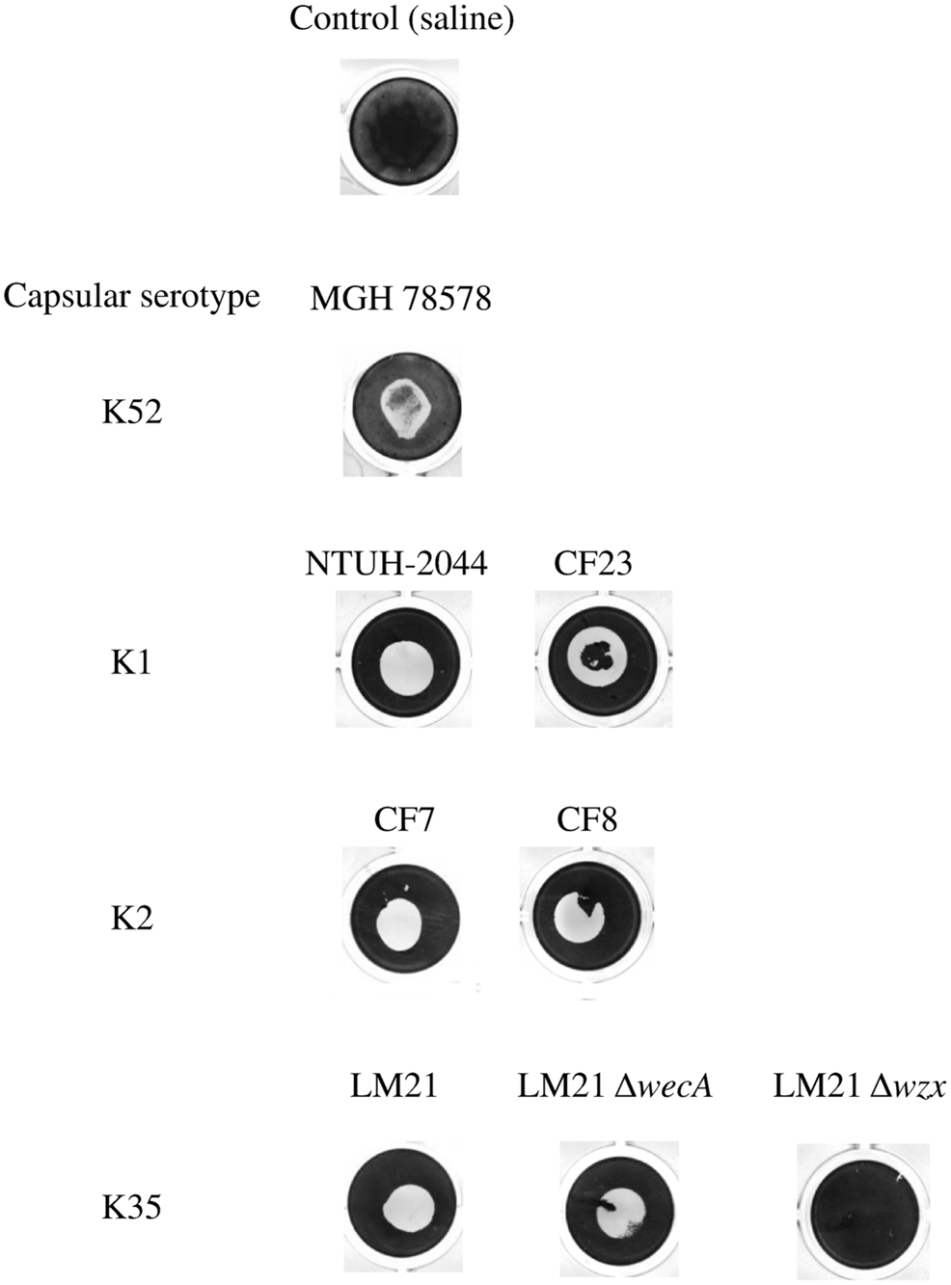
Inhibition of biofilm formation by surface coating with *K. pneumoniae* CPS extracts. Inhibition of *S. epidermidis* biofilm formation in polystyrene microtiter plate wells after coating the surface with CPS extracts from several *K. pneumoniae* strains (wild-type strains of different serotypes, capsule and LPS deficient mutants of strain LM21) or saline (control).

To investigate the mode of action of the anti-biofilm component, we used capsular extracts of *K. pneumoniae* MGH 78578 (serotype K52), but from also strains belonging to other capsular serotypes: K1 (NTUH-2044 and CF23), K2 (CF7 and CF8) and K35 (LM21). Previous coating of these CPS extracts onto the surface of polystyrene wells efficiently repelled *S. epidermidis* biofilm formation, (Fig. 7), indicating that the CPS inhibits the bacteria-surface interactions of the abiotic substrate, whatever the capsular serotype. A similar phenotype was observed with extract from a LPS O-antigen deficient mutant of LM21 strain (LM21Δ*wecA*) whereas extracts from its capsule isogenic deficient strain (LM21Δ*wzx*) did not show any inhibition (Fig. 7), indicating that capsule extract but not LPS O-antigen was responsible for the anti-biofilm phenotype.

## Discussion

*Klebsiella pneumoniae* is a widespread Enterobacteria, which lives in surface water, soil, on plant and as commensal resident of the mammalian nasopharynx and gastrointestinal tract. Its capacities to form biofilm have been largely investigated [28]–[32] and most of the isolates are heavily surrounded by capsular exopolysaccharides [33], [34]. In this study, we isolated and characterized a *K. pneumoniae* exopolysaccharide with anti-biofilm activity against both Gram-positive and Gram-negative bacteria. Several bacteria produce polysaccharides with anti-biofilm activity, such as *Escherichia coli*, *Pseudomonas aeruginosa*, *Lactobacillus acidophilus*, *Bacillus licheniformis*, *Streptococcus phocae*, *Kingella kingae*, *Vibrio sp.* and *Actinobacillus pleuropneumoniae* [9], [10], [19]–[22], [35]–[37]. These polymers, mainly composed of glucose, galactose, mannose, glucuronic acid or rhamnose, are either CPS components such as Ec111, Ec300 of *E. coli* and the CPS (serotype 5) of *A. pleuropneumoniae*, or non capsular polysaccharides such as the PAM galactan and the Pel/Psl molecules produced by *K. kingae* and *P. aeruginosa* PAO1, respectively. As with *K. pneumoniae* in this study, most of the previously described anti-biofilm polysaccharides were detected in both planktonic culture supernatants and in biofilm extracts [18], [19] indicating that this synthesis was more related to the cells density than to the sessile condition. The higher inhibition observed in our study with planktonic supernatant compared to biofilm extracts (Fig. 2A) is likely due to differences both in the amount of biological material in the initial samples and in the recovery procedures.

HPAEC analysis of dried supernatant from *K. pneumoniae* showed that the polysaccharide with anti-biofilm activity was composed of five monosaccharides (galactose, glucose, rhamnose, glucuronic acid and glucosamine), three of them, rhamnose, galactose and glucose, being specific of some *K. pneumoniae* CPS structures previously characterized [38]. In order to determine if the active macromolecules in *K. pneumoniae* supernatant extract were indeed CPS,^ 1^H NMR analysis (Fig. 5) was carried out and confirmed, based on the comparison with other NMR analysis of CPS described in literature [39], the presence of capsular polysaccharide structure in the dried extract. Indeed, the assignment of the ^1^H NMR analysis was very similar to that of the capsular polysaccharide reported by Stenutz *et al.*, with a resolved resonance in the anomeric region (4.5–5.5 ppm) and chemical shifts at 1.27 and 1.41 ppm, which is the characteristic signal of methyl protons of rhamnose residue ([→2)-*α*-l-Rha*p*-(1→]; [→4)-*α*-l-Rha*p*-(1→] respectively [39]. The signals in the anomeric region at 4.58 ppm, 5.21 ppm and 5.45 ppm have been attributed to galactose type sugar residues and more especially to [→3)-*β*- d-Gal*p*-(1→], [*α*-d-Gal*p*-(1→] and →2,3)-*α*- d-Gal*p*-(1→] residues respectively [39]. Therefore, it is likely that the structure of the polysaccharide isolated in this study from *K. pneumoniae* MGH 78578 (K52 serotype) dried extract is similar to the CPS [39]. However, slight differences were observed. As described, the hydrolysis of K52 capsular polysaccharides with TFA gave rise to galactose and rhamnose with the molar ratio of 3∶2 [39], whereas in the case of *K. pneumoniae* MGH 78578 dried extract, a molar ratio of 3∶1 was observed. Moreover, the HPAEC analysis revealed the presence of additional sugars such as glucose and glucosamine compared to the published K52 CPS composition. The presence of these additional sugars could be attributed to contamination by the culture medium (glucose) and by traces of LPS fraction. Indeed, as described by Kubler-Kielb *et al*. (2013), many LPS cores from *K. pneumoniae* are composed of glucosamine residues (GlcN) [38]. Despite these differences, further characterization of the polydispersity and purity performed by SEC-MALLS analysis clearly indicated that the polysaccharides from *K. pneumoniae* MGH 78578 dried extract possessed high molecular mass and was therefore more likely capsular polysaccharides. Indeed, capsular polysaccharides such as amylovoran from *Erwinia amylovora*, or stewartan from *Pantoea stewartii* have been previously evaluated to be in the range of 1–2 MDa [40].

Several capsular polysaccharides have been associated with anti-biofilm activities. Karwacki *et al.* recently showed that anti-biofilm activities of *Actinobacillus pleuropneumoniae* are due to capsular polysaccharide [37]. Some group 2 capsular polysaccharides of *E. coli*, composed of simple polysaccharides, also exhibited non-biocidal anti-biofilm activity [9], [19]. The *E. coli* capsules classification is based on their structure, organization and mechanisms of biosynthesis [41] and *K. pneumoniae* capsules are related to the *E. coli* group 1 capsule. These polymers are acidic, possess a low charge density and do not make colonic acid. To our knowledge, no study had yet demonstrated the anti-biofilm activity of this group of polysaccharides. The *K. pneumoniae* CPS anti-biofilm activity was not dependent on the capsular serotype, since capsular extracts from serotypes K1, K2, K35 and K52 exhibited this activity (Fig. 7). In addition and although traces of LPS could be present in capsule extracts, the role of LPS O-antigen was ruled out since capsule extract from a *wecA* deficient mutant showed anti-adhesion properties similar to that of the wild-type strain (Fig. 7). Nethertheless the role of other components of the LPS such as the core polysaccharide cannot be ruled out.

Several hypotheses have been formulated regarding the mechanism of action of anti-biofilm polysaccharides. Most of them inhibit biofilm formation by coating the abiotic surfaces and therefore modifying the initial adhesion of the bacteria to the substrate [9], [19], [37]. Some of them also impaired the bacterial aggregation, as described by Karwacki *et al.* [37]. In our study, precoating of surface with CPS extract indicated that *K. pneumoniae* CPS probably inhibits the *S. epidermidis* bacteria-surface interactions rather than disrupting the bacterial interactions. This was supported by the fact that incubation of a 6 h old *S. epidermidis* biofilm with *K. pneumoniae* CPS extract did not modify the biomass after 2 h of further incubation, compared to a control biofilm without any CPS addition (data not shown). We hypothesize that CPS modifies the physical properties of abiotic surfaces by increasing its hydrophobicity. However, differences were observed between isolates within the *S. aureus* species, owing probably to differences in individual bacterial cell surface characteristics. Using a genetic approach, Travier *et al.* recently showed that modifications in surface physicochemical properties of *E. coli* cells were able to modify the anti-biofilm activity of group 2 anti-biofilm capsule polysaccharides, probably due to changes in ionic charge and Lewis base properties induced by the CPS polysaccharides [42].

We previously showed that the capsular polysaccharide of *K. pneumoniae* is implied in surface adhesion, spacing and ordering of bacteria in the initial step of biofilm formation, and is required for late biofilm maturation step ([31]; Muraglia *et al*. unpublished data). Though the initial adhesion of bacteria on the surface constitutes a key step in the formation of biofilm, the spread of bacteria on the surface is also another important factor in the formation of a biofilm, especially in a multispecies highly competitive environment. Some Gram-negative rods such as the highly motile *Pseudomonas aeruginosa* produce exopolysaccharides that promote their own surface movement during the early stages of biofilm formation [43]. *K. pneumoniae* is a non motile Gram-negative rod and may have developed different social strategies leading to surface exclusion of competitors by large CPS production and therefore allowing successful surface colonization.

## Supporting Information

Figure S1 **Dose-dependent effect of** ***K. pneumoniae*** **supernatant.** *S. epidermidis* (inoculum, 10^6^ CFU/mL) biofilm formation was measured in the presence of several concentrations of *K. pneumoniae* supernatant with the BioFilm Magnetic bead aggregation assay®: without supernatant (•); with undiluted supernatant (⧫); with supernatant diluted to 1/2 (▴); supernatant diluted to 1/5 (▪) and supernatant diluted to 1/10 (*). Quantitative data were expressed as BFI (means ± SD of three determinations) and the dotted line represents the threshold of magnetic bead-mediated agglutination detection.
(TIF)
